# Cervicothoracic flap for complex facial defect reconstruction: a case based approach

**DOI:** 10.1080/23320885.2025.2607239

**Published:** 2025-12-22

**Authors:** Frank Andrés Álvarez Vásquez, Andres Camilo Herrera, Luis Arturo Molina, Ivan Enrique Rodriguez Mantilla

**Affiliations:** ^a^ Doctor in Medicine, Universidad del Rosario, Bogotá, Colombia; ^b^ Department of Epidemiology, Universidad El Bosque, Bogotá, Colombia; ^c^ Independent Plastic and Reconstructive Microsurgeon, Fundación Universitaria de Ciencias de la Salud, Bogotá, Colombia

**Keywords:** Cervicothoracic flap, head and neck surgery, oncologic resection, reconstructive surgery

## Abstract

Reconstruction of complex facial defects following oncologic resection presents a significant challenge, especially when critical anatomical subunits and nerve branches are involved. The choice of reconstructive technique must prioritize tissue match, reliability, and safety. The cervicothoracic fasciocutaneous flap offers a valuable alternative to microsurgical reconstruction, providing robust vascular supply and favorable aesthetic integration without the need for microsurgical techniques. This report details the case of a 76-year-old male with micronodular basal cell carcinoma of the left cheek. Wide oncologic resection resulted in an 8 × 8 cm defect involving the infraorbital, preauricular, nasogenian, and mandibular units, as well as underlying structures including the SMAS and masseteric fascia. The defect was successfully covered using the cervicothoracic flap, with no complications such as necrosis or local infection. At four-month follow-up, the patient exhibited stable healing, good aesthetic integration, and only mild residual ectropion, with no functional limitations or donor site morbidity. This case highlights the effectiveness of cervicothoracic flaps in comorbid patients, positioning them as a viable alternative to microsurgical reconstruction for complex facial defects.

## Introduction

The oncologic resection of extensive facial lesions represents a significant reconstructive challenge for the plastic surgeon. Beyond restoring cutaneous coverage, reconstruction must also consider facial symmetry, neuromuscular function, and overall aesthetic integration. These factors become particularly relevant in elderly individuals or patients with significant comorbidities—such as hypertension and controlled type 2 diabetes mellitus—who may not tolerate prolonged operative times or microvascular free-flap reconstruction [1,2].

Multiple reconstructive strategies have been described for head and neck defects, including skin grafting, local flaps, primary closure, pedicled regional flaps, and free tissue transfer. Although free flaps remain the gold standard for large and complex defects, their use requires specialized microsurgical expertise, institutional availability of equipment, prolonged operative time, and favorable patient characteristics that reduce the risk of vascular compromiso [1–3].

The cervicothoracic fasciocutaneous flap represents a reliable alternative for patients unsuitable for microvascular reconstruction. Its robust vascular supply and skin characteristics similar in color and thickness to cervicofacial tissues—allow for adequate coverage of large defects [4,5]. Despite its usefulness, the technique remains relatively underutilized, and its reconstructive potential may be underestimated in contemporary practice [6,7].

**Figure 1.**
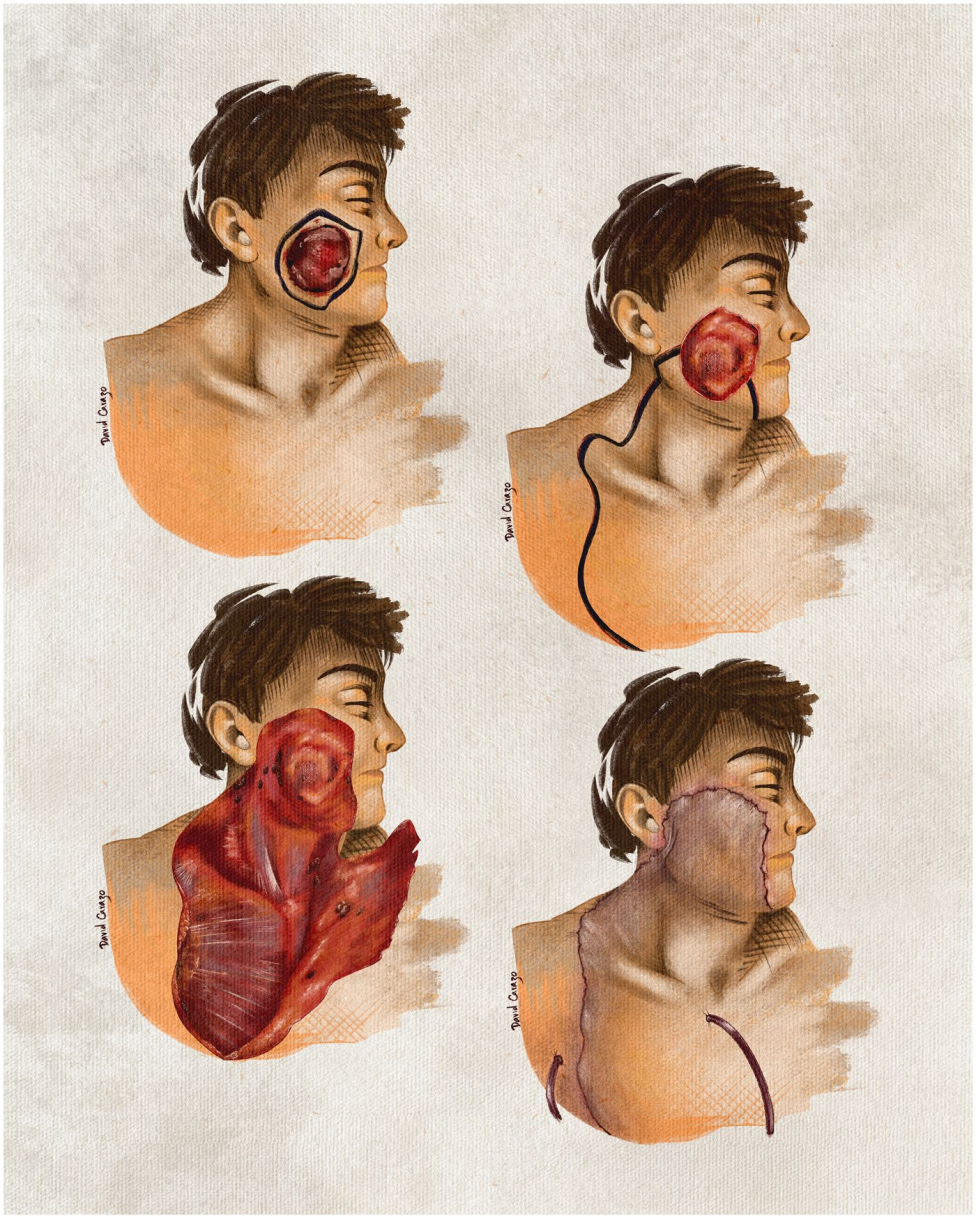
Schematic representation of the surgical sequence: oncologic resection, resulting defect, cervicothoracic flap design and elevation, defect coverage, and final reconstruction.

We present the case of a man with hypertension and controlled type 2 diabetes mellitus who underwent wide oncologic resection for micronodular basal cell carcinoma, resulting in an extensive defect of the left cheek. Reconstruction was performed using a cervicothoracic fasciocutaneous flap, achieving satisfactory functional and aesthetic outcomes with minimal morbidity (Figure 1). This case reinforces the relevance of this flap as a safe and efficient option, particularly when microsurgical reconstruction is not ideal due to patient or institutional constraints.

## Clinical case

**Figure 2.**
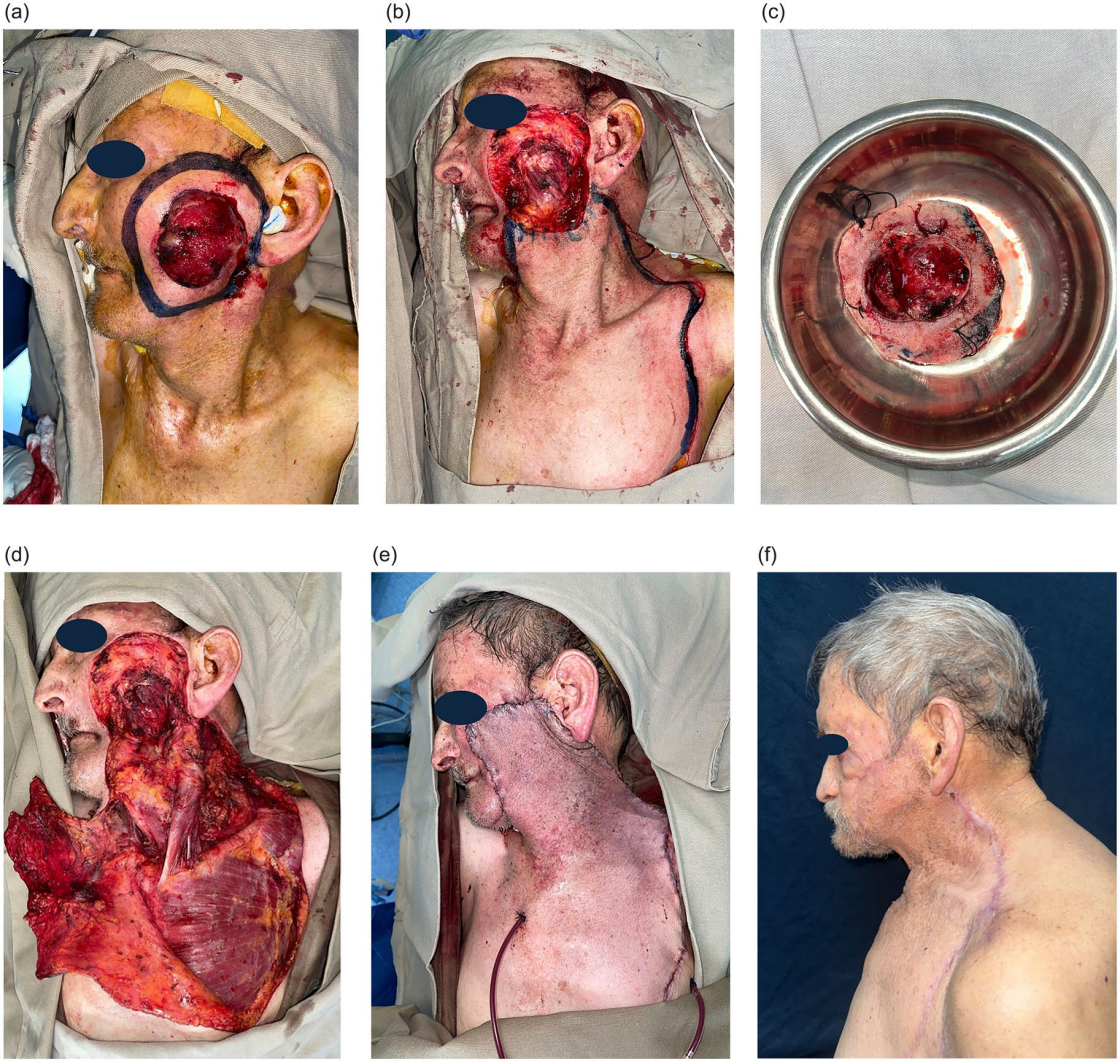
(a) Ulcerated, exophytic 6 × 6 cm lesion involving the left cheek and mandibular subunits, with active bleeding. (b) Post-resection defect measuring 8 × 8 cm after wide excision involving multiple facial subunits. (c) Pathologic specimen submitted for analysis. (d) Preoperative design of the cervicothoracic fasciocutaneous flap. (e) Intraoperative view showing flap inset with suspension to the maxilla. (f) Four-month postoperative outcome demonstrating satisfactory coverage and mild ectropion.

A 76-year-old man with a medical history significant for hypertension and controlled type 2 diabetes mellitus presented with a 3-year history of a progressively enlarging, exophytic 6 × 6 cm lesion on the left cheek and mandibular región (Figure 2a). The lesion showed ulceration, active bleeding, and perilesional trophic changes. Histopathology confirmed micronodular basal cell carcinoma (Figure 2b).

The head and neck surgery team performed wide local excision with 2 cm margins, resulting in an 8 × 8 cm defect compromising the left infraorbital, preauricular, cheek, nasogenian fold, and mandibular subunits. Resection extended to the masseteric fascia and the SMAS, with involvement of the temporofacial and cervicofacial branches of the facial nerve (Figure 2c).

Reconstruction was performed using a cervicothoracic fasciocutaneous flap (Figure 2d). A meticulous dissection of the transverse cervical and thoracoacromial perforators was performed according to anatomical planes. The flap was then advanced and inset into the defect, achieving full coverage. Layered closure was performed using Vicryl and Monocryl sutures. To decrease the risk of postoperative ectropion, the flap was suspended to the maxilla using supportive sutures. The donor site was closed primarily without tensión (Figure 2e).

Postoperatively, the flap survived completely without partial or total necrosis. No hematoma, infection, or wound dehiscence occurred. At 4-month follow-up, the patient demonstrated good overall aesthetic and functional outcomes, with only mild residual lower eyelid ectropión (Figure 2f).

## Discussion

Reconstruction of extensive cervicofacial defects continues to represent a formidable challenge within reconstructive surgery, particularly in geriatric patients or those burdened with systemic comorbidities such as arterial hypertension and controlled type 2 diabetes mellitus, conditions that compromise microcirculatory integrity, reduce physiologic reserve, and significantly diminish tolerance for prolonged anesthetic exposure and microvascular operative times. Since the foundational description of the cervicothoracic fasciocutaneous flap by Garrett in 1966, subsequently refined by Kaplan, this regional flap has been consistently validated as a robust, versatile, and anatomically predictable option for the restoration of extensive defects involving the midface, lateral cheek, mandibular contour, preauricular region, parotid bed, and upper cervical soft tissues [1–3]. Its thick, pliable, hair-bearing skin provides an excellent color, texture, and thickness match for facial reconstruction, surpassing distant donor sites and avoiding the donor-site morbidity intrinsic to free flap harvest [4]. Although free tissue transfer—particularly the anterolateral thigh, radial forearm, scapular/parascapular, and fibula osteocutaneous flaps—remains the gold standard for complex, three-dimensional reconstructions, its feasibility is frequently undermined in elderly individuals due to cumulative endothelial dysfunction, diabetes-related microangiopathy, decreased arterial compliance, higher prevalence of atherosclerotic burden, and increased susceptibility to anastomotic thrombosis [5,6]. Multiple contemporary series report elevated failure rates (6%–8%) in patients older than 70 years or those with significant systemic disease, accompanied by longer ICU stays, increased cardiopulmonary complications, and prolonged global hospitalization, all of which reinforce the necessity of selecting reconstructive strategies proportionate to the patient’s physiologic resilience [6–8].

The cervicothoracic fasciocutaneous flap derives its perfusion from a dense plexus of perforators arising from the internal thoracic, suprascapular, and superficial cervical arterial systems, facilitating wide arc of rotation, reliable distal perfusion, and predictable survival even in large flaps extending 20–22 cm [2,3,6]. This flap avoids microvascular anastomosis entirely; therefore, despite the utility of optical magnification and fine instrumentation to enhance pedicle dissection, it does not constitute microsurgical reconstruction [7]. This distinction is critical for institutions with limited microsurgical capability, as the flap can be executed safely under loupe magnification or, in experienced hands, without magnification at all [8]. Nonetheless, like all cervicofacial advancement flaps, it is associated with well-described complications including distal epidermolysis or partial necrosis (reported in 5%–12% of cases), hypertrophic scarring—particularly in Fitzpatrick IV–VI phenotypes—restricted cervical mobility secondary to broad subplatysmal undermining, tension-related midfacial distortion, and lower-eyelid malposition, with ectropion representing the most characteristic and physiologically predictable drawback due to inferior vector traction on the flap and lateral canthal dehiscence or attenuation.

With respect to ectropion management, contemporary literature emphasizes the necessity of both intraoperative prophylaxis and structured postoperative correction. Preventive strategies such as maxillary periosteal suspension, lateral canthopexy, temporofascial anchoring, and midface vertical suspension have demonstrated superior capacity to neutralize the downward gravitational vector exerted by cervicofacial or cervicothoracic flaps [9]. In a recent retrospective analysis, larger defect dimensions, reconstructive modality using cervicofacial-type flaps, and the requirement for multidisciplinary oncologic involvement were independently associated with increased ectropion incidence, underscoring the biomechanical susceptibility of the lower eyelid in this reconstructive context [10]. Furthermore, modern surgical algorithms classify ectropion into involutional, paralytic, cicatricial, and mechanical subtypes, advocating for nuanced interventions such as combined medial and lateral canthopexies, tarsal strip procedures, myocutaneous suspension, full-thickness skin grafting, and orbicularis oculi myocutaneous flaps. Notably, laterally based orbicularis oculi myocutaneous flaps have demonstrated high efficacy in cases of recurrent or persistent ectropion by providing dynamic muscular support, improving anterior lamella integrity, and restoring lower-eyelid position without significant morbidity [11]. In our case, the development of mild residual ectropion despite appropriate prophylactic suspension sutures aligns with published outcomes and reinforces the importance of integrating biomechanical, anatomical, and patient-specific factors when planning reconstruction.

When evaluating alternative reconstructive strategies, free flaps remain advantageous for defects requiring bulk restoration, osseous reconstruction, or complex tridimensional contouring; however, in elderly patients or those with hypertension, diabetes, or previous smoking history, their physiological costs may outweigh their reconstructive benefits. By contrast, locoregional flaps—including cervicofacial, cervicothoracic, supraclavicular artery island flaps, and pectoralis major myocutaneous flaps—offer shorter operative durations, reliable vascularity, reduced donor-site morbidity, and greater reproducibility in centers without access to microsurgical infrastructure. When flap length exceeds 22 cm or when pivot-point angulation becomes excessive, distal ischemia risk increases significantly, warranting consideration of free flap transfer as a safer alternative. In our patient, the cervicothoracic flap demonstrated excellent vascular reliability, full viability, harmonious aesthetic integration, and minimal complication burden, reaffirming its role as a highly valuable, resource-efficient, and physiologically appropriate reconstructive strategy for elderly patients with comorbidities such as hypertension and controlled diabetes mellitus, and establishing its continued relevance as a cornerstone in the reconstructive surgeon’s armamentarium.

## Conclusion

The cervicothoracic fasciocutaneous flap is a safe, practical, and effective reconstructive option for extensive cervicofacial defects, especially in elderly patients or those with comorbidities such as hypertension and diabetes mellitus that make microvascular reconstruction less desirable. Although magnification facilitates pedicle dissection, this technique does not require microsurgical anastomosis, making it suitable for institutions without microsurgical resources. Surgeons should remain vigilant for complications such as ectropion and distal necrosis and implement preventive strategies accordingly. This case underscores the value of the cervicothoracic flap as a resource-efficient, reliable method providing satisfactory functional and cosmetic outcomes in complex facial reconstruction.
